# Expression of Concern: MicroRNA-208a-3p functions as an oncogene in colorectal cancer by targeting PDCD4

**DOI:** 10.1042/BSR-2018-1598_EOC

**Published:** 2022-11-10

**Keywords:** Colorectal cancer, micrRNA-208-3p, PDCD4

The Editorial Office has been made aware of potential issues surrounding the scientific validity of this paper, hence has issued an expression of concern to notify readers whilst the Editorial Office investigates. It is noted that similarities exist between the Figure 7A PDCD4 western blots and the Caprin1 blots from Figure 5B of Yu et al. 2020 (doi: 10.1007/s13205-020-02433-9). Similarities also exist between the Figure 3A HCT116 miR-208a-3p inhibitor panel and the miR-302c mimics panel of Figure 3A from Wu et al. 2019 (doi: 10.1042/BSR20190421), and between Figure 2D HCT116 Inhibitor NC panel and the Figure 2G mimics NC panel of the same paper, Wu et al. 2019 (doi: 10.1042/BSR20190421). The Editorial Office has also identified similarities between the western blot panels from figure 4D, where the PDCD4/HCT116 band appears to be horizontally flipped for the PDCD4/SW480 bands.

